# How Do Environmental Concerns and Governance Performance Affect Public Environmental Participation: A Case Study of Waste Sorting in Urban China

**DOI:** 10.3390/ijerph18199947

**Published:** 2021-09-22

**Authors:** Hang Yin, Yixiong Huang, Kuiming Wang

**Affiliations:** 1 School of International and Public Affairs, Shanghai Jiao Tong University, Shanghai 200030, China; yin_hang@sjtu.edu.cn; 2 Department of Economics, Law and Society, ESSCA School of Management, EU-Asia Institute, 49003 Angers, France; yixiong.huang@essca.fr; 3 China Institute for Urban Governance, Shanghai Jiao Tong University, Shanghai 200030, China

**Keywords:** environmental protection, waste sorting, public participation, urban governance, governance performance, China

## Abstract

Environmental pollution threatens public health and has become a social concern in recent years. Despite the conditions for public participation in environmental governance have improved considerably, the level of public engagement in government projects still falls short of expectations. Therefore, this article introduced two key variables, hoping to answer the following research question that how environmental concerns and governance performance affect public environmental participation. Through principal component analysis of the data from the “Survey of Chinese Urban Residents’ Attitudes toward Environmental Protection”, the findings of this article are as follows: First, public environmental concerns have no significant impact on their environmental engagement; second, the improvement of residents’ confidence in the government performance of environmental management reduces their willingness to participate in official projects. The higher the confidence in the government’s performance, the lower the level of public engagement is. Moreover, due to the consideration of self-interest or lack of environmental awareness, those who oppose waste incineration in waste terminal disposal tend to take a non-participatory role in waste sorting programs. Therefore, we suggest that the government have more diverse shareholders in environmental protection, so it should expand public participation through education, publicity, mobilization, and incentives.

## 1. Introduction

Since the 1980s, the rise of civil society has led to a greater diversity of stakeholders in social governance. The state or the government is no longer the only dominant party in social governance, and the will and opinion of the public are becoming increasingly important. Thus, in recent years, public participation has become increasingly critical in urban environmental governance [1,2,3,4]. In particular, due to excessive growth of domestic waste and disputes over disposal methods, such as waste incineration and landfills, public health has been threatened and many popular protests have even been triggered. The government alone cannot cope with all environmental challenges, and public engagement and support can facilitate the achievement of environmental protection goals. Compared with state-led environmental projects, voluntary public participation has two advantages. From the public’s point of view, their environmental participation can help residents better understand and discover environmental problems; from the perspective of social governance, public participation can provide a bottom-up approach and experiences for environmental protection [5]. In addition, public participation can share the increasing operational cost of urban environmental protection and relieve the financial burden of government expenditures [6,7].

In developing countries such as China, the start of public participation is regarded as an important transitional point of urban environmental governance [8]. Both the government and scholars believe that public participation can complement the traditional state-led model of environmental protection [9] and can respond in a timely manner to a variety of urban ecological problems [10]. Moreover, active communities can cultivate residents’ sense of identity [11] and achieve successful collective actions [12]. Therefore, public participation in environmental management is crucial to the Chinese government. This paper aims to explore the effects of public environmental concerns and governance performance on public participation in official environmental protection through empirical data analysis on waste sorting schemes in China to provide approaches and suggestions for the implementation of official policies in achieving the improvement of environmental quality goals.

China is also facing unique practical public participation challenges in its socioeconomic and cultural context. Accelerating urbanization has gradually destroyed the social network that originated in agrarian society, which is essential to collective actions. People living in urbanized communities come from different geographical locations and have diverse regional cultures, so they lack mutual understanding and trust. For them, it is difficult to form a common social identity to carry out collective actions. Thus, the government’s leadership has become a choice for China to mobilize public involvement under its authoritarian system. The legitimacy and authority of the Chinese government are mainly based on its governance performance. Therefore, residents’ recognition of environmental protection also comes from the performance of the government. In other words, local governments’ appeal for public participation depends on residents’ evaluation of the environmental quality and the impact of state-led environmental protection programs.

Regrettably, the lack of enough attention to urban environmental problems in the early years [13] led to the slow response of the government [14] to the ineffectiveness of environmental governance [15]. As a result, the government’s authority in environmental management was seriously weakened, indicating that it is difficult for them to motivate the public to participate in official programs [16]. In response to the public’s environmental appeals, China has been investing numerous resources in environmental protection and has significantly improved the quality of air, water, and urban environment [17,18,19,20]. Data show that from 2007 to 2019, Chinese government investment in environmental protection increased from CNY 99.58 billion to CNY 744.4 billion, an increase of nearly 7.5 times [21,22]. The number of cities with high PM 2.5 levels dropped from 221 in 2015 to 126 in 2017, and the number of residents exposed to air pollution decreased from 75% to 49.2%. Meanwhile, the proportion of seriously polluted waters in the country’s seven major revers decreased from 27% in 2005 to 8.3% in 2017 [23,24].

Consequently, the citizens widely recognize the encouraging achievements in environmental protection. According to the longitudinal environmental survey of “Chinese Urban Residents’ Attitude towards Environmental Protection” by Shanghai Jiao Tong University, 64.65% of the respondents in 2019 believed that China’s ecological environment continues to improve, a 24.46% increase compared to the 2017 data. Meanwhile, the percentage of people who said that the environment harmed their health dropped from 51.15% to 32.92%. Moreover, public satisfaction with the municipal government’s performance in environmental governance rose from 53.1% in 2013 to 77.0% in 2019.

Unexpectedly, the increased public satisfaction has not increased their environmental participation, which is reflected in the official waste sorting initiative to be discussed in this paper. Most of the existing literature was carried out under the background of poor government performance, and the continuous deterioration of the environment has led to a lack of public awareness of environmental protection and a lack of participation [13,16]. In contrast, this paper is carried out under the background of improving the performance of government environmental governance.

## 2. Literature Review

This section mainly reviews the policy changes of the Chinese government on municipal waste treatment and proposes hypotheses about the factors affecting public participation in environmental governance based on the existing literature.

### 2.1. History of Urban Waste Disposal Change in China

In the 1990s, rapid urbanization brought sudden population growth to China’s major cities. The expansion of the urban population has produced a large amount of domestic waste, which is far beyond the capability of existing waste disposal facilities. Generally speaking, landfills and incineration are the most economical ways to dispose of waste from the government’s perspective, but due to the harm to the ecological environment and public health, they have been widely resisted and protested by the public. On the one hand, excessive landfills threaten water and soil safety, resulting in an increase in chronic diseases among nearby residents [25,26]. On the other hand, high carcinogens such as dioxins and residues produced by waste incineration also endanger public health [27,28]. People living near incinerators have a higher rate of cancer than the general population [29].

Thus, in 2000, eight major cities including Beijing and Shanghai introduced waste sorting pilot programs to address the challenge of urban waste disposal. This initiative has two practical implications for the government. First, waste sorting can reduce the harm to public health caused by traditional extensive incineration or landfill processes [30]. Second, waste sorting can not only improve the efficiency of waste treatment but also reduce its cost, indicating that the number and speed of the construction of waste treatment facilities can be slowed down [31].

However, at that time, environmental issues were neither a policy priority in government nor a public concern, so the initiative quickly fizzled. The failure of the initiative from the government’s point of view is related to inadequate preparation. For example, pilot cities have dissimilar standards for waste classification, and back-end disposal facilities are also seriously insufficient [32]. For the public, although waste sorting can gradually improve the environment, it will also cause many inconveniences to daily life. Moreover, the benefits of waste sorting cannot directly improve the living environment of residents, so public participation is relatively low [33]. In China, the government has long been the only stakeholder of social governance, so the public has only expressed verbal support for waste sorting but has little practical action.

In the end, the government opted for a more practical and manageable waste incineration scheme [34]. Nevertheless, with the growth of public income and environmental awareness, in recent years, the construction of waste incineration and landfill facilities has been greatly challenged. Public concerns about the safety of waste incineration facilities and government mistrust eventually led to a series of “Not in my backyard” (NIMBY) environmental protests, with many incineration plans shelved and some facilities shut down. Since 2009, more than half of all public environmental protests in China have been related to establishing waste incineration facilities [35]. Many mass protests have even threatened the government’s authority and resulted in environmental accountability issues [36,37,38].

As a result, in 2019, the government had to reconsider domestic waste sorting to cope with the fast growth of urban waste and the lack of disposal capacity. In July of the same year, a nationwide waste sorting scheme was proposed. Having learned lessons from previous trouble caused by waste incineration plants, municipal governments have adopted four methods to regain the public’s trust and persuade them to participate in recycling: cleaning up residues in incineration settings, building recycling infrastructure, training sanitation workers, and setting up detailed rules and penalties for waste sorting in advance [39,40]. Moreover, the implementation of environmental regulation and strict supervision in recent years have not only improved living conditions in urban cities but also helped the government regain public trust. Municipal governments hope to take this opportunity to encourage the public to actively participate in waste sorting.

The implementation of the waste sorting initiative has the following impacts on residents. Shanghai was the first city in China to implement mandatory waste sorting, and other cities have largely followed its rules [41]. First, residents are required to classify waste into four categories according to local waste sorting regulations: kitchen waste, recyclable waste, hazardous waste, and residual waste. Second, residents are required to throw away waste bags at designed times, limiting their flexibility in the daily schedule. Third, residents need to dump domestic waste at a fixed location, so most of the waste stations and bins in the community compounds have been closed or removed. Locals have to spend more time and walk more distance to complete the waste drop. Since the process of waste sorting is complicated and time-consuming, the actual participation of the Shanghai public fails to reach the expected goal at the early stage [42]. The government has to hire sanitation workers and volunteers to supervise residents’ waste-dropping conduct at garbage stations [43].

In summary, China’s urban environmental governance, particularly the urban waste disposal process, indicates that residents’ environmental concerns and the government’s environmental governance performance may affect public engagement in environmental protection [44]. On the one hand, disappointing environmental quality and ineffective governance performance hinder public participation in official programs. On the other hand, the refinement of environmental quality and government achievements also cannot correspondingly encourage public participation.

### 2.2. Research Hypothesis 

#### 2.2.1. Public’s Environment Concerns

Public environmental concerns are essential determinants affecting people’s willingness for environmental involvement [45]. Residents usually make their participation choices based on assessments of factors such as air and water quality, as well as the city’s overall pollution levels and hazardous risks [46]. These aspects together constitute public environmental concerns [47]. Since the evaluation of environmental status requires professional knowledge [48] and the public does not have specialized ability, their perception is mostly rough and inaccurate [49,50]. Thus, residents regularly cognize the surrounding environment to decide whether to take part in environmental activities. Public perceptions of environmental status are therefore often at odds with professional judgment. Some researchers believe that public concerns about environmental risks could trigger popular protests.

China’s urban governance process indicates that undesirable environmental quality and long-lasting environmental pollution are major obstacles to public participation in official programs and the induction of NIMBY protests. However, other studies suggest that the public does not care about environmental quality or the existence of risk factors, and they use it more as an excuse to engage in mass protests [51]. Thus, the improvement of environmental quality does not necessarily motivate residents to participate in official programs. Moreover, since the public’s perception of environmental quality is relatively abstract, it is insufficient to initiate their environmental actions. From this, the study hypothesizes as follows:

**Hypothesis** **1** **(H1).** *Public environmental concerns do not affect residents’ willingness to participate in official environmental protection projects*.

#### 2.2.2. Governance Performance

Governance performance is one of the important sources of governmental legitimacy in China [52], which also applies to environmental protection practices [53]. If the government wants to gain public recognition and participation in the government’s programs, it first needs to improve the governance performance of environmental protection. Unsatisfying governance performance not only causes damage to the government’s authority and makes people less likely to respond to its call for public participation [54] but also triggers environmental protests. Taking the urban waste problem as an example, it was the insufficient preparation and poor performance of the government that iced residents’ enthusiasm as well as participation willingness in previous waste sorting pilots. After that, the slow response of the municipal government to NIMBY protests caused by waste incineration plants triggered severe social unrest. In short, public dissatisfaction with the government’s performance weakens the government’s authority and discourages residents from participating in official projects [55].

Similarly, the government’s efforts and performance in improving environmental protection can rebuild its credibility and attract public support [56]. However, as mentioned above, the will did not transform into actual support. The comparison between residents’ strong willingness and actual passive attitude indicates that the government’s achievements in environmental governance did not always raise the public’s willingness to participate in environmental programs. This phenomenon is different from the theoretical expectation.

One explanation of such phenomena is that weak self-governance and strong state-led tradition make people heavily reliant on the government. For residents, better environmental governance performance means that it is less necessary to be involved in environmental programs. The public’s increasing satisfaction with and dependence on the government’s environmental governance makes them believe that they do not need to participate in promoting waste sorting. In short, people’s willingness to participate decreases with the improvement of governance performance. Thus, the corresponding hypothesis is as follows:

**Hypothesis** **2** **(H2).** *An improvement in government environmental performance may diminish the willingness of the public to participate in official environmental protection projects*.

#### 2.2.3. Comparison of Different Waste Disposal Approaches

Currently, environmental pollution and risk are some of the main motivations of street protests in China. Statistics show that more than half of all mass protests in China from 2000 to 2013 were related to environmental pollution [57]. Rather than experts‘ and officials’ emphasizing the efficiency of waste disposal, public protests are directly related to the environmental risks and pollution around their residential compounds such as building landfills or incineration facilities [58]. However, in the case of urban raising waste and insufficient disposal capacity, the government and the public need to choose between waste sorting/reprocessing and waste incineration. On the government side, the rapidly growing number of mass protests in China has to some extent threatened social stability and government authority. Subsequently, the government had to stop incineration projects and rechoose the waste sorting option. As the turning point in urban waste management, the waste incineration protest movement changed the government’s environmental policy preference. On the public side, many opinion leaders and the public have also expressed support for the waste sorting option [59,60].

There are three advantages of waste sorting over waste incineration. First, the results of waste sorting are obvious to residents, while they cannot assess the harm caused by waste incineration [61]. Second, waste sorting is more environmentally friendly than waste incineration [62]. Lastly, waste sorting costs less to operate for the government because residents share the fiscal burden on the government [63]. Choosing waste incineration or waste sorting is a “pick one from two” problem for residents; thus, they are more willing to accept the waste sorting option to avoid becoming uncertain minority victims of waste incineration [64].

However, the public may not accept either waste incineration or waste sorting options, because they are unwilling to bear the cost of urban waste disposal. On the one hand, the construction of waste incineration facilities has caused many popular protests in China because of the threat to their health [65]. That is, the public bears the cost of environmental management. On the other hand, the public also shares the cost of waste sorting management as it causes a lot of inconvenience to residents’ life, such as consuming extra living and working time. Since many protests against waste incinerations enforced governments to cancel or suspend the waste incineration projects in the past decades, the public may also oppose waste sorting programs for the reason to reduce the burden of such practice.

Thus, the corresponding hypothesis is as follows:

**Hypothesis** **3** **(H3).** *Residents who oppose waste incineration are less likely to take part in waste sorting programs*.

## 3. Materials and Methods

### 3.1. The Data Source

The data used in this article are from the “Survey of Chinese Urban Residents’ Attitudes toward Environmental Protection” conducted by Shanghai Jiao Tong University in 2019. The survey covered a total of 35 major cities nationwide, including municipalities, provincial capitals, and other second-tier cities. Through the computer assisted telephone interview system (CATIS), 100 questionnaires were collected in each city, for 3500 samples. According to their landline number or mobile phone number, all the respondents were randomly polled by the CATIS and interviewed by experienced investigators. Diversified samples generated at random by the CATIS can better reveal the overall status of residents involved in waste sorting programs.

The survey data consist of four parts: respondents’ demographic information, environmental concern measurements, governance performance measurements, and attitudes on different environmental protection approaches. Among them, respondents’ attitude toward waste sorting is the dependent variable, and other measurements are independent variables. In addition, cities are introduced as a variable to control for potential effects at the urban level. The main measures of this study are presented in Table 1 (excluding demographic measures).

Residents’ demographic information included gender (male, female), age (<18, 18–29, 30–39, 40–49, 50–59, >60), educational achievement (primary school, junior high school, senior high school, college degree, bachelor’s degree, master’s degree, doctor’s degree), and income level (low income, relatively low income, middle income, relatively high income, high income). Additionally, all respondents were native residents or permanent residents (at least five years of residence), so their perceptions of environmental concern and governance performance can be credible.

Citizens’ attitudes toward waste incineration plants and waste sorting are the two key variables of this study because attitudes toward waste incineration may affect their willingness to participate in waste sorting. The descriptions in the questionnaire were as follows: “To what extent do you accept the construction of a waste incineration plant near your residence?” and “Would you like to take part in waste sorting programs?”. A four-point Likert scale measured both questions. Higher scores mean stronger acceptance or willingness. The answers were recorded before inclusion in the analysis.

People’s perception of local environmental status and municipal governments’ performance in environmental protection are major study determinants. The measures of environmental concerns include perception of air safety, water safety, and overall environmental quality. Meanwhile, the respondents’ willingness to take environmental action measures such as traveling on public transport, avoiding the use of disposable products, willingness to donate to environmental NGOs, and becoming volunteers were also investigated.

The evaluation of governments’ performance in environmental protection includes the transparency of environmental policies and projects, municipal government’s environmental protection performance, and the respondents’ confidence in local/central governments’ future performance in environmental protection. All questions were measured by a four-point Likert scale (higher scores mean better evaluation).

### 3.2. Methods and Linear Model

There are two levels of variables in the study: individual and city. At the individual level, the point is extracting variables from specific measurements, and at the city level, it is important to control the effect of unknown factors.

As mentioned above, in the questionnaire, a variety of measurements were used to measure respondents’ environmental concerns and their evaluation of governance performance. Although the questions are designed based on existing research, it is difficult to determine whether the public’s actual perception is in line with the questionnaire. Hence, it is necessary to test the internal consistency of the measurements and then extract variables with dimension reduction methods such as principal component analysis.

Significant regional differences in China indicate that unknown factors may heavily affect the analysis results at the city level. Cities’ gross domestic product (GDP), environmental protection expenditure, and environmental status all have an impact on the public’s environmental concerns, as well as on residents’ willingness to participate in government programs. However, since it is almost impossible to determine key potential factors at the city level and collect the data accurately, this study only introduces cities as a control variable.

According to the analysis above, the linear model is as follows:

Y waste sorting = α + β1 × F1 + β2 × F2 + β3 × x waste incineration opposition + θ × X + γ + ε

As presented above, the dependent variable, Y waste sorting, is the respondents’ willingness to participate in waste sorting programs. F and β are the components extracted by principal component analysis and their coefficients, X are covariates included demographic variables, α is the intercept, γ is the confirmed effect of surveyed cities, and ε is the error term.

## 4. Results

### 4.1. Demographics

**Figure 1:**
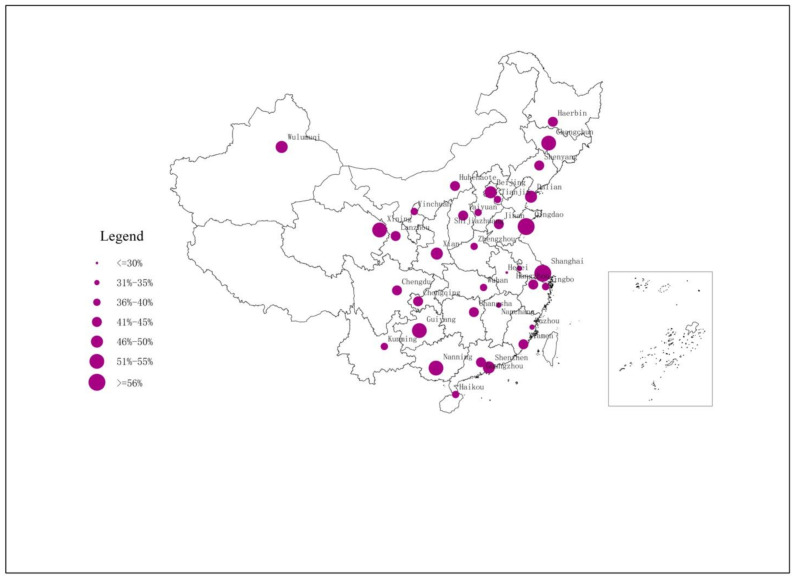
Percentage of waste sorting strong supporters in China’s main cities.

The demographic data of the survey are summarized in Table 2. Thirty-five hundred respondents included 1928 men (55%) and 1580 women (45%). The survey data show a high level of support among Chinese residents for government waste sorting programs (Figure 1). More than half of the urban residents expressed a strong willingness to support the waste sorting program.

The survey results of the residents’ attitudes toward waste incineration and waste sorting are as expected. A total of 68.9% of residents are unwilling/reluctant to accept waste incineration in their community. Meanwhile, 41.7% of respondents supported waste sorting or at least did not oppose it (52.9%), and only approximately 5% of respondents refused to participate in waste sorting programs. In general, the public dislikes waste incineration due to its potential environmental risk and prefers environmentally friendly choices such as waste sorting.

### 4.2. Principal Component Analysis and Variables Extracting

The principal component analysis is used to extract variables from respondents’ environmental concerns and to evaluate governance performance in environmental protection programs. The measurements are grouped by their internal consistency, and measurements that do not have enough internal consistency are filtered. There are three measurements of environmental concerns (respondents’ evaluation of water quality and food safety, governance performance in environmental programs) and two measurements of governance performance (the exception of central/local government performance). Their Cronbach’s α is shown in Table 3.

Through the principal component analysis of the data, we find that environmental concerns and governance performance are two different principal components for the public. Considering that the improvement of environmental quality in recent years is closely related to the government’s action and investment [23,24], such results are noteworthy. According to Table 3, the measurement “Governance Performance” is included in the component of “Environmental Concern.” In contrast, the should-be component “Government Performance” is replaced by “Public Confidence in Local/Central Government’s Performance.” It shows that, for the public, confidence in the government is more significant than the government’s performance. In other words, achievements in environmental protection make most residents optimistic toward environmental governance, even though the reality is not as ideal as expected.

### 4.3. The Impact of Public Environmental Concerns and Governance Performance on Public Participation Willingness

The linear model consists of public environmental concerns, governance performance, public participation willingness, and other control variables, including demographics and cities (Table 4). Public participation willingness in waste sorting programs is the dependent variable. Models 1 and 3 introduce environmental concerns, public confidence in government performance, and respondents’ attitudes toward waste incineration. Model 3 is the full model, which contains all components and variables.

According to the results in Table 4, environmental concerns have no significant effect on respondents’ willingness to participate in environment protection (*p*-value = 0.805). In contrast, residents’ confidence in governance performance has a significant effect on public engagement (*p*-value = 0.000). The value of adjusted R2 increases by the numbers of variables that were included in the regression model, which explains the small value of adjusted R2 in our model. Meanwhile, the respondents’ attitude toward waste incineration also affected their willingness to participate in the waste sorting program (*p*-value = 0.000). In summary, public environmental concerns did not affect the public’s environmental engagement, and the public’s increased confidence in government and their attitude toward waste incineration harmed their collective participation. In other words, all three hypotheses are verified.

Among the three determinants, the effect of the public’s confidence in government is the most significant (coefficient value = −13.69), and the coefficient of “Attitude Toward Waste Incineration” is −7.35, which is relatively lower than the public confidence. Therefore, the data show that public confidence in the performance of government environmental management is the most effective determinant. In addition, public environmental concern’s effect on respondents’ willingness to participate is weaker than expected. Although significantly impacts the dependent variable, the “Attitude Toward Waste Incineration” mechanism still needs further explanation.

## 5. Discussion

In general, the analysis results indicate that in addition to public environmental concerns, governance performance and respondents’ attitudes toward waste incineration both have a significant negative effect on public participation in official environmental protection. Such results demonstrate that most residents rely heavily on the government for environmental protection and take a passive attitude toward public participation. In other words, if citizens think their municipal government can effectively deal with environmental problems, their willingness to participate then decreases. The details of different determinants are as follows:

### 5.1. The Explanation of Public Environment Concerns’ Insignificant Effect

Existing research suggests that public environmental concern motivates residents to take collective action [66]. However, according to the results of the principal component analysis, residents’ environmental concerns are mostly scattered and can only agree in some very specific respects. As the results indicate, through the questionnaire including approximately 15 measures of respondents’ environmental perception and behavior, only three measures underwent the test of internal consistency. It seems that the public’s environmental concerns consist of micro-level measures, such as the perception of water safety, food safety, and the evaluation of local government performance. Macro-level measures, for instance, overall environmental quality, are mostly filtered. Moreover, none of the measures of environmental behavior passed the test. These results illustrate those current public environmental concerns are concentrated on a few micro-level aspects, and most residents do not know how to carry out collective actions to respond to the environmental crisis.

Since citizens’ environmental concerns are almost have nothing to do with collective environmental actions, they can hardly contribute to public participation in environmental protection. The consequence is that people do not take any measures unless they are facing serious environmental risks, such as waste incineration projects. Nonetheless, existing environmental pollution and risks may still be the excuses of passive attitudes toward environmental programs, such as waste sorting and NIMBY protests, so the improvement of environmental quality still has significance in environmental protection.

### 5.2. The Effect of Public Confidence in Government

Governance performance is considered to be the most significant determinant of residents’ environmental participation [67]. However, it is important to point out that the public’s confidence in governance performance, not the governance performance itself, is the actual determinant. In other words, the increase in people’s confidence is unrelated to their perception and evaluation of the environment. The public’s confidence and trust in the government’s environmental governance largely come from recognizing China’s economic success [53,68]. In this case, the public will be dependent on the government and “give up” their role as environmental participants and stakeholders. Therefore, the enthusiasm of the public to engage in waste sorting programs will be weakened.

This circumstance also reflects that the public is still deeply influenced by the traditional administrative model; that is, the government is the only and dominant stakeholder of social governance, so environmental protection is the responsibility of the government, and the public does not play an important role in environmental management. Such a phenomenon also has a certain danger. First, the public is not aware of the negative impact of extensive waste disposal methods on the environment and themselves. Second, the public’s “blind” trust in the government leads to their over-reliance on the government in environmental management, which will eventually become an obstacle for the public to participate in waste sorting. Third, if the government cannot fulfill the public’s expectations, then the authority and legitimacy of the government will face a collapse. Eventually, it could lead to popular protests that threaten the social stability the government cares about.

### 5.3. The Effect of Waste Incineration

The data results also indicate that those who oppose waste incineration tend to take a non-participating role in waste sorting programs. Such a conclusion is unexcepted and counterintuitive. For rational individuals, or the “economic man,” waste sorting is not only more acceptable in common sense but also a choice according to economic rationality [69]. First, the urban waste problem is inevitable during the urbanization process, and every individual must share the governance cost in society. Second, for rational citizens, the risk from waste incineration plants is unpredictable, while the cost of avoiding such risk (for instance, moving away) is sometimes unaffordable. Lastly, resisting waste incineration is widely accepted and even supported, but it may be regarded as improper and selfish as opposed to waste sorting.

These findings certainly have substantial implications for the government’s environmental management. In the eyes of the public, it is wrong for the government to build waste incineration plants because it harms the health of residents. Therefore, the mass protest against waste incineration construction is both reasonable and legitimate. However, waste sorting is widely recognized as an environmentally friendly measure, so residents who oppose it have no legitimate reason to take collective actions. Moreover, people who are unwilling to engage in waste sorting programs normally do not have a strong awareness of environmental protection. Not only will they oppose initiatives that harm their health, but they will also be passive about measures such as recycling that increase household chores. In contrast, the public with a high awareness of environmental protection will actively engage in waste sorting and may even compromise in waste incineration projects.

Residents with high environmental awareness pay attention to environmental protection and actively participate in waste sorting programs, while those lacking environmental awareness tend to take a non-participating role. To consider their self-interests, residents who lack environmental awareness may resist anything unfavorable for them. For example, non-participants reject incineration facilities because they are environmentally dangerous. They choose not to engage in waste sorting because they lack the time and energy to do so. This is why those who oppose waste incineration in waste terminal disposal are fiercer and more likely to be passive in official projects.

## 6. Conclusions

In conclusion, the findings of this study suggest that the improvement of the public’s governmental protection performance exceptions diminishes their willingness to participate in environmental programs. In contrast, people’s environmental concerns have little effect. Due to the consideration of their self-interest or lack of environmental conscientiousness, those who oppose waste incineration in waste terminal disposal tend to take a non-participating role in waste sorting programs. Such results reveal two important findings. First, the public still lacks the motivation and experience to participate in collective actions. Second, the public relies heavily on the government to shoulder the responsibility of social governance alone. However, with the rapid growth of urban waste in recent years, the public has become more aware of the environmental challenges related to their health and has taken collective action to address their interests. Although the public still wants the government to take full responsibility for waste disposal, this is not a realistic option, because modern environmental governance cannot be achieved without public participation. Therefore, encouraging public engagement in environmental protection should be the priority of the government in environmental governance.

From the perspective of public management, although the conclusion of the data shows that the improvement of environmental quality and management performance cannot increase public participation, the government cannot “give up” the efforts of environmental management. Neglecting to address environmental challenges may trigger public discontent that threatens the government’s leadership and legitimacy. Therefore, the improvement of the government’s environmental governance performance may not be able to meet the growing expectation of the public. Based on this, the authors believe that the government should encourage residents to participate in social–environmental protection initiatives by employing publicity, mobilization, and incentives. As a stakeholder of environmental management, the public is not only the beneficiary but also bears the responsibility of promoting environmental protection.

Feasible regulations and supervision are essential to public participation in waste sorting and other environmental programs. Thus, we believe that there are three steps to construct a similar long-term motivating mechanism in China. First, the government should clarify residents’ rights and obligations in environmental protection programs by creating regulations. Second, supervision and punishment are necessary to avoid the “broken window phenomenon” in public participation. Third, the regulations and scale of supervision should be adjusted promptly to adapt to the circumstances of public participation. In sum, the empirical study on China’s waste sorting program suggests that alleviating public environmental concerns and raising the exception of environmental performance diminish people’s willingness to participate in environmental protection. Introducing public participation as an assistance of the traditional state-led model can be challenging. A regular supervision mechanism is necessary for promoting public participation in environmental protection in China.

In conclusion, this study focuses on factors that have changed significantly in China in recent years namely environmental quality, governance performance, and different disposal methods, based on China’s conventional top-down governing structure. In terms of future research, the public’s time availability, social class, waste sorting rules, the location of waste facilities, incentives for waste sorting, and individual literacy of residents, can be discussed later.

## Figures and Tables

**Table 1:** Measures.

	Measures	Description
**The willingness of waste sorting**		Would you like involving in the waste sorting program? (”Supportive” “Neutral” “Reluctant” “Unwilling”)
**Environmental** **Overall Status Evaluation**	**Local environmental quality**	The degree on local environment quality (1~10 point scale, degree of quality increases with the score)
	**Local water safety**	The degree on local water safety (1~10 point scale, degree of safety increases with the score)
	**Local food safety**	The degree in local food safety (1~10 point scale, degree of safety increases with the score)
	**Local pollution risk**	The degree of local pollution’s risk (”Serious” “Normal” “Mild” “None”)
**Environmental** **Governance Performance Evaluation**	**Environmental information disclosure**	The degree of the municipal government’s performance in environmental information disclosure (1~10 point scale, disclosure level increases with the score)
	**Governance performance**	Evaluation of local government’s environmental performance (1~10 point scale, performance increases with the score)
	**Public Confidence in the Performance of the Central Government**	Confidence in local government’s environmental performance (“Very high” “High” “Normal” “Low”)
	**Public Confidence in the Performance of the Local Government**	Confidence in local government’s environmental performance (“Very high” “High” “Normal” “Low”)
**Other Potential Determinants**	**Public transportation using frequency**	How often do you use environmental-friendly transportation, like a bike or bus? (“Very often” “Often” “Occasionally” “Never”)
	**Recycled product using frequency**	How often do you choose recycled products? (“Very often” “Often” “Occasionally” “Never”)
	**Respondents’ willingness to donate to environmental protection projects**	Would you like to donate to an environmental NGO or projects? (“Very willing” “Willing” “Reluctant” “Unwilling”)
	**Respondents’ participation willingness of environmental protection projects**	Would you like to take part in environmental programs or become a volunteer? (“Very willing” “Willing” “Reluctant” “Unwilling”)
**The opposition of Waste Incineration Plants**		To what extent do you accept the construction of a waste incineration plant near your residence? (”Completely Accept” “Accept” “Reluctantly Accept” “Unacceptable”)

**Table 2:** Demographics and citizens’ attitude toward waste sorting/incineration.

Variables	Categories	Number of Respondents	Percentage
**Gender**	Male	1928	55%
	Female	1580	45%
**Age**	18~29	1231	35.1%
	30~39	1086	31.0%
	40~49	700	20.0%
	50~59	349	9.9%
	≥60	142	4.0%
**Educational Achievement**	Primary School	93	2.7%
	Junior high school	207	5.9%
	Senior high school	607	17.3%
	College Degree	803	22.9%
	Bachelor’s degree	1498	42.7%
	Master’s degree	241	6.9%
	Doctor Degree	59	1.7%
**Income Level**	Low Income	298	8.5%
	Relative Low Income	924	26.3%
	Middle Income	1915	54.6%
	Relative High Income	324	9.2%
	High Income	47	1.3%
**Attitude Toward Waste Incineration**	Complete Accept	152	4.4%
	Acceptable	922	26.7%
	Reluctantly Accept	1449	41.9%
	Unacceptable	935	27.0%
**Attitude Toward Waste Sorting**	Support	1464	41.7%
	Neutral	1854	52.9%
	Reluctant	153	4.4%
	Unwilling	37	1.1%

**Table 3:** The components of the principal analysis.

Components	Measurements	Cronbach’s α
**Environmental Concerns**	Evaluation of Water Quality	0.702
	Evaluation of Food Safety	
	Governance Performance	
**Public Confidence in Local/Central** **Government’s Performance**	Public Confidence in the Performance of the Central Government	0.778
	Public Confidence in the Performance of the Local Government	

**Table 4:** Linear model of public environmental concern, governance performance, and public participation willingness (waste sorting).

	Model 1	Model 2	Model 3
**Environmental Concerns**	−2.16 ** (0.038)	0.27 (0.785)	−0.25 (0.805)
**Public Confidence in** **Government’s Performance**		−13.95 *** (0.000)	−13.69 *** (0.000)
**Attitude Toward Waste Incineration**			−7.35 *** (0.000)
**Gender**	−5.68 *** (0.000)	−5.09 *** (0.000)	−4.85 *** (0.000)
**Age**	−2.76 *** (0.009)	−2.27 *** (0.030)	−1.54 (0.133)
**Educational Achievements**	−5.29 *** (0.000)	−5.61 *** (0.000)	−5.38 *** (0.000)
**Income Levels**	−3.01 *** (0.005)	−3.91 *** (0.000)	−3.81 *** (0.001)
**Intercept**	35.11 *** (0.000)	35.20 *** (0.000)	37.63 *** (0.000)
**Confirm Effect of City**	Controlled		
**N**	3508	3508	3508
**Adjusted R^2^**	0.016	0.042	0.048

** *p* < 0.05 *** *p* < 0.01.

## Data Availability

For data on the “Survey of Chinese Urban Residents’ Attitudes towards Environmental Protection,” please contact the corresponding author.
